# The macular inner plexiform layer thickness as an early diagnostic indicator for Parkinson’s disease

**DOI:** 10.1038/s41531-022-00325-8

**Published:** 2022-05-25

**Authors:** Xin Wang, Bin Jiao, Xiaoliang Jia, Yaqin Wang, Hui Liu, Xiangyu Zhu, Xiaoli Hao, Yuan Zhu, Bei Xu, Sizhe Zhang, Qian Xu, Junling Wang, Jifeng Guo, Xinxiang Yan, Beisha Tang, Rongchang Zhao, Lu Shen

**Affiliations:** 1 Department of Neurology, Xiangya Hospital, Central South University, Changsha, China; 2 National Clinical Research Center for Geriatric Disorders, Central South University, Changsha, China; 3 Engineering Research Center of Hunan Province in Cognitive Impairment Disorders, Central South University, Changsha, China; 4 Hunan International Scientific and Technological Cooperation Base of Neurodegenerative and Neurogenetic Diseases, Changsha, China; 5 Key Laboratory of Hunan Province in Neurodegenerative Disorders, Central South University, Changsha, China; 6 School of Computer Science and Engineering, Central South University, Changsha, China; 7 Health Management Center, the Third Xiangya Hospital, Central South University, Changsha, Hunan China; 8 Eye Center of Xiangya Hospital, Central South University, Changsha, China; 9 Key Laboratory of Organ Injury, Aging and Regenerative Medicine of Hunan Province, Changsha, China

**Keywords:** Parkinson's disease, Parkinson's disease, Diagnostic markers

## Abstract

Whether structural alterations of intraretinal layers are indicators for the early diagnosis of Parkinson’s disease (PD) remains unclear. We assessed the retinal layer thickness in different stages of PD and explored whether it can be an early diagnostic indicator for PD. In total, 397 [131, 146, and 120 with Hoehn-Yahr I (H-Y I), H-Y II, and H-Y III stages, respectively] patients with PD and 427 healthy controls (HCs) were enrolled. The peripapillary retinal nerve fiber layer (pRNFL), total macular retinal thickness (MRT), and macular volume (TMV) were measured by high-definition optical coherence tomography, and the macular intraretinal thickness was analyzed by the Iowa Reference Algorithms. As a result, the PD group had a significantly lower average, temporal quadrant pRNFL, MRT, and TMV than the HCs group (all *p* < 0.001). Moreover, the ganglion cell layer (GCL), inner plexiform layer (IPL), and outer nuclear layer were thinner in patients with PD with H-Y I, and significantly decreased as the H-Y stage increased. In addition, we observed that GCL and IPL thicknesses were both correlated with Movement Disorder Society-Unified Parkinson’s Disease Rating Scale III (MDS-UPDRS III) scores and non-motor symptoms assessment scores. Furthermore, macular IPL thickness in the superior inner (SI) quadrant (IPL-SI) had the best diagnostic performance in patients with PD with H-Y I versus HCs, with a sensitivity and specificity of 75.06% and 81.67%, respectively. In conclusion, we confirmed the retinal structure was significantly altered in patients with PD in different clinical stages, and that GCL and IPL changes occurred during early PD disease and were correlated with MDS-UPDRS III scores and non-motor symptoms assessment scores. Furthermore, macular IPL-SI thickness might be performed as an early diagnostic indicator for PD.

## Introduction

Parkinson’s disease (PD), characterized by selective loss of nigral dopaminergic neurons, is the second most common neurodegenerative disorder^1^. Apart from predominant motor symptoms, visual disturbances, such as impaired visual acuity, visuospatial dysfunction, and visual hallucinations, can also be observed in patients with PD^2^. The retina, an extension of the central nervous system, has attracted significant attention in the brain pathological processes of neurodegenerative disorders in recent years^3^. Dopamine-containing amacrine cells play a role in integrating visual information. Their dendritic branch extends from the retinal inner nuclear layer (INL) to the inner plexiform layer (IPL), where it is connected to bipolar cells and retinal ganglion cells^4^. Studies have shown that the immunoreactivity of tyrosine hydroxylase in dopaminergic cells^5^ and the retinal dopamine content in PD are decreased^6,7^. In addition, there are phosphorylated α-synuclein and Lewy body deposits in the retina of patients with PD^8^. These observations suggest that retinal pathological changes probably occur in patients with PD.

The diagnosis of PD at an early stage remains challenging. High-definition optical coherence tomography (HD-OCT), a noninvasive optical imaging technique that allows cross-sectional retinal microstructure imaging quantitatively, has become an appealing candidate imaging modality in PD. Many previous studies have explored the alterations in retinal morphology of patients with PD in vivo using OCT imaging^9–13^. However, whether alterations in the intraretinal structure in patients with PD can act as an indicator for early diagnosis of PD remains unclear. Thus, a random eye of all participants in our study was assessed by HD-OCT, and macular intraretinal segmentation was further analyzed using advanced automated three-dimensional (3D) retinal layer segmentation software (Iowa OCTExplore version 3.8.0)^14^. In addition, we evaluated the potential association between retinal measures and clinical parameters in PD and explored the most sensitive and specific retinal indicator for the early diagnosis of PD by plotting a receiver operating characteristic (ROC) curve.

## Results

### Characteristics of study participants

**Fig. 1:**
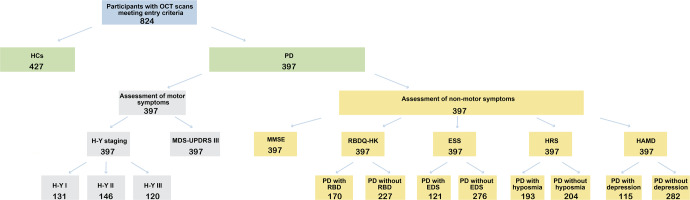
Flow diagram of participant selection and study design. OCT optical coherence tomography, HCs healthy controls, PD Parkinson’s disease, H-Y Hoehn-Yahr, MDS-UPDRS III Movement Disorder Society-Unified Parkinson’s Disease Rating Scale part III, MMSE Mini-Mental State Examination, RBD rapid eye movement sleep behavior disorder, RBDQ-HK RBD questionnaire-Hong Kong, ESS Epworth sleepiness scale, EDS excessive daytime sleepiness, HRS Hyposmia Rating Scale, HAMD Hamilton rating scale for depression.

A total of 824 participants, including 397 patients with PD (131 Hoehn-Yahr [H-Y] stage I, 146 H-Y II, 120 H-Y III) and 427 healthy controls (HCs), were included in the present study between December 2017 and November 2019 (Fig. 1). There were no statistically significant differences in age, sex, intraocular pressure (IOP), and best-corrected visual acuity (BCVA) between the PD and HCs groups. There were also no significant differences among the PD subgroups regarding age at onset, sex, IOP, and BCVA. The PD with H-Y III stage had higher age, disease duration, and Movement Disorder Society-Unified Parkinson’s Disease Rating Scale III (MDS-UPDRS III) scores compared to the other groups. The relevant clinical and demographic characteristics of all participants are listed in Table 1.

**Table 1:** Clinical and general demographics characteristics of all the participants.

Variables	All participants		*p* values	PD			*p* values
	HCs	PD, total		H-Y I stage	H-Y II stage	H-Y III stage	
Number	427	397	–	131	146	120	–
Demographics							
Age, y	61.50 ± 7.46	62.30 ± 7.68	0.128^b^	60.58 ± 7.28	62.51 ± 7.81	63.93 ± 7.63	**0.002**^**c**^
Age of onset	–	56.58 ± 7.96	–	55.83 ± 7.26	56.77 ± 7.59	57.18 ± 9.07	0.224^d^
Sex, (m/f)	219/208	192/205	0.401^a^	63/68	77/69	52/68	0.310^a^
Duration	–	5.72 ± 3.50	–	4.76 ± 3.03	5.73 ± 2.96	6.75 ± 4.24	**<** **0.001**^**c**^
Assessment of motor symptoms, Mean (P_25_, P_75_)							
Hoehn-Yahr	–	2 (1, 3)	–	–	–	–	–
MDS-UPDRS III	–	23 (15, 36)	–	14 (11, 24)	23.5(17, 34)	35 (24, 47)	**<** **0.001**^**d**^
Assessment of non-motor symptoms, Mean (P_25_, P_75_)							
MMSE	–	28 (25, 29)	–	28 (26, 30)	28 (25, 29)	27 (24, 29)	**0.002**^**d**^
RBDQ-HK	–	13 (3, 32)	–	8 (2, 21)	14 (3, 35)	21 (6, 37)	**<** **0.001**^**d**^
ESS	–	6 (2, 12)	–	5 (2, 9)	7 (3, 12)	8 (3, 15)	**0.033**^**c**^
HRS	–	23 (12, 24)	–	24 (16, 24)	18 (12, 24)	22 (12, 24)	**0.025**^**c**^
HAMD	–	4 (0, 8)	–	2 (0, 7)	3 (0, 8)	5 (2, 10)	**0.002**^**c**^
Ophthalmologic parameters							
IOP (mmHg)	15.63 ± 3.03	15.93 ± 2.75	0.140^b^	15.90 ± 2.76	15.87 ± 3.01	16.01 ± 2.43	0.918^c^
BCVA, Snellen	1.20 ± 0.25	1.19 ± 0.31	0.478^d^	1.22 ± 0.31	1.18 ± 0.29	1.17 ± 0.33	0.372^c^

Significant results appear in bold.

*PD* Parkinson’s Disease, *HCs* healthy controls, *MDS-UPDRS III* Movement Disorder Society-Unified Parkinson’s Disease Rating Scale III, *P*_*25*_ 25% percentile, *P*_*75*_ 75% percentile, *y* years, *MMSE* Mini-Mental State Examination, *RBDQ-HK* Rapid Eye Movement sleep behavior disorder (RBD) questionnaire-Hong Kong, *ESS* Epworth sleepiness scale, *HRS* Hyposmia Rating Scale, *HAMD* Hamilton rating scale for depression, *IOP* intraocular pressure, *BCVA* best-corrected visual acuity.

^a^Pearson’s *χ*2 test.

^b^Student’s *t*-test.

^c^One-way ANOVA.

^d^Non-parametric test.

### Peripapillary retinal nerve fiber layer and macular parameters in patients with Parkinson’s disease and healthy controls groups

**Fig. 2:**
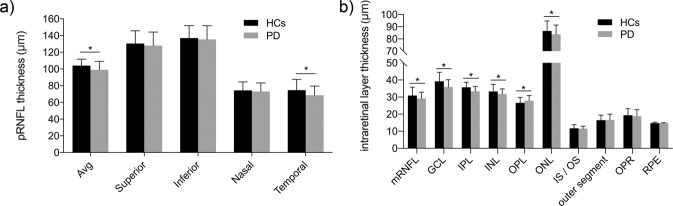
Comparison of the retinal parameters between the Parkinson’s disease (PD) and healthy controls groups. **a** The average and temporal quadrant pRNFL thicknesses were significantly lower in the PD group than in the HCs group. **b** Patients with PD had a thicker OPL and thinner mRNFL, GCL, IPL, INL, and ONL. The error bars the indicate standard deviation of PD or HCs. HCs healthy controls, PD Parkinson’s disease, pRNFL peripapillary retinal nerve fiber layer, mRNFL macular retinal nerve fiber layer, GCL ganglion cell layer, IPL inner plexiform layer, INL inner nuclear layer, OPL outer plexiform layer, ONL outer nuclear layer, IS/OS inner segment/outer segment, OPR photoreceptor outer segment/retinal pigment epithelium, RPE retinal pigment epithelium.

**Fig. 3:**
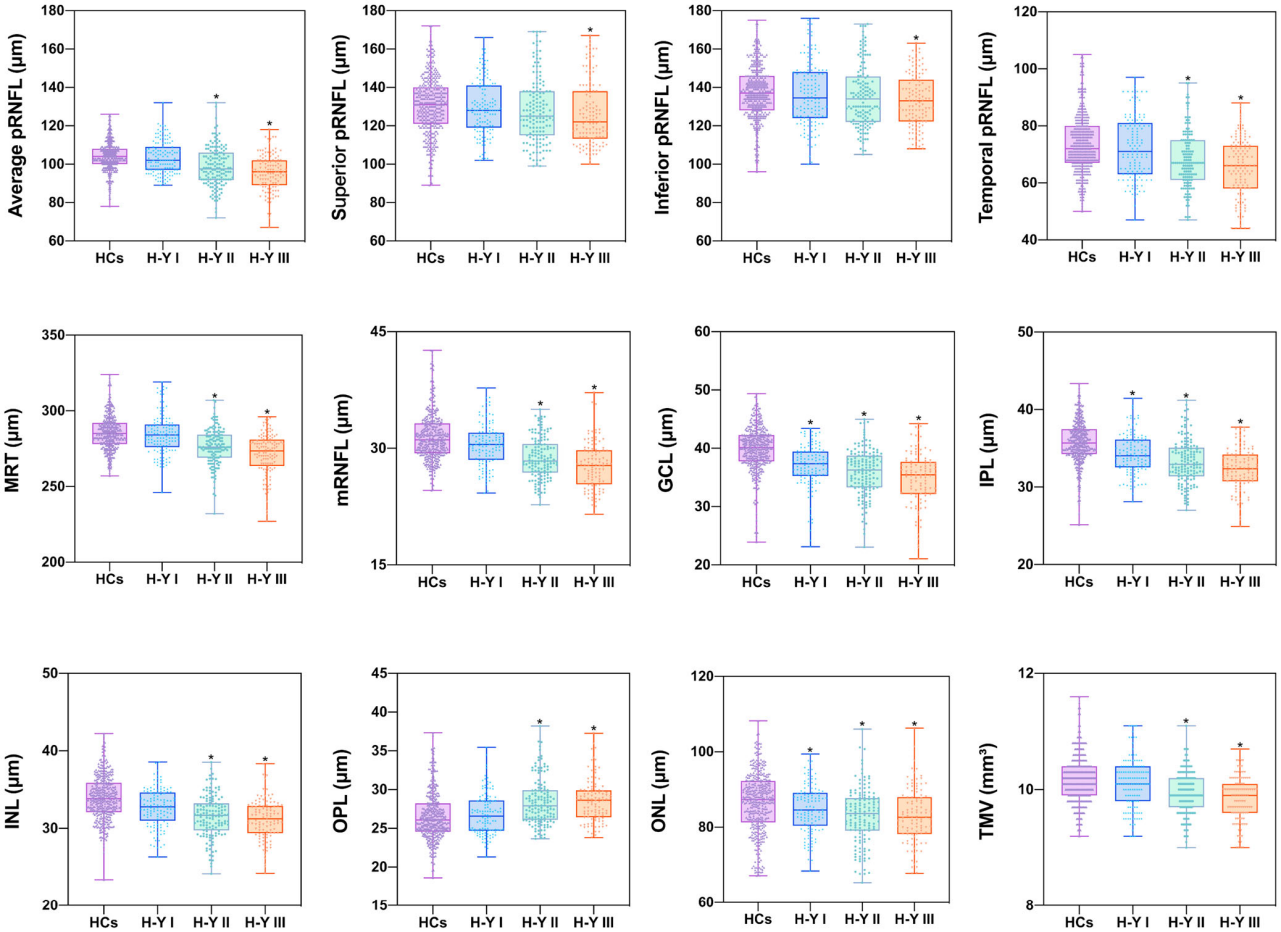
The retinal parameters in patients with Parkinson’s disease at different clinical stages versus healthy controls. Patients with PD had a thicker OPL and thinner mRNFL, GCL, IPL, INL, and ONL. These tendencies were more pronounced with higher Hoehn-Yahr staging scores. In addition, significant thinning of the GCL, IPL, and ONL could be detected early in patients with PD with the H-Y I stage. Box-and-whisker plots represent the median (bar), interquartile (box), min and max values (whiskers). HCs healthy controls, PD Parkinson’s disease, H-Y Hoehn-Yahr, pRNFL peripapillary retinal nerve fiber layer, MRT macular retinal thickness, mRNFL macular retinal nerve fiber layer, GCL ganglion cell layer, IPL inner plexiform layer, INL inner nuclear layer, OPL outer plexiform layer, ONL outer nuclear layer, TMV total macular volume.

The generalized linear model showed the mean and temporal quadrant peripapillary retinal nerve fiber layer (pRNFL) thicknesses were significantly lower in the PD group than in the HCs group (*p* < 0.05) (Fig. 2). Further analysis showed abnormal changes in pRNFL thickness in the temporal quadrant could be detected in patients with PD with the H-Y II stage. Additionally, patients with the H-Y III stage had significantly lower superior and inferior quadrant pRNFL thickness compared to the HCs (Fig. 3). This indicated that pRNFL thickness was preferentially reduced in the temporal quadrant, followed by the superior and inferior quadrants, and finally, may be extended in the nasal quadrant.

In terms of macular morphological changes, most of the intraretinal layer thicknesses showed statistical differences between the PD and HCs groups. We found that, compared with the HCs, the total macular volume (TMV) (9.92 ± 0.44 mm^3^ in patients with PD vs 10.17 ± 0.43 mm^3^ in HCs) and macular retinal thickness (MRT) (277.8 ± 12.39 μm in patients with PD vs 285.2 ± 12.40 μm in HCs) demonstrated statistically significant decreases in patients with PD. Patients with PD showed a thicker outer plexiform layer (OPL) and thinner macular retinal nerve fiber layer (mRNFL), ganglion cell layer (GCL), IPL, INL, and outer nuclear layer (ONL) (Fig. 2). In addition, we observed all macular intraretinal layer thicknesses [other than outer plexiform layer (OPL)] decreased, and OPL increased as the H-Y staging score increased (Fig. 3). Furthermore, significant thinning of the GCL, IPL, and ONL thickness could be detected early in patients with PD with the H-Y I stage. In addition to the above intraretinal layers, we found that significant mRNFL and INL thinning, and OPL thickening occurred in patients with PD with H-Y II and H-Y III stages (Fig. 3).

### Association between optical coherence tomography parameters and motor symptoms

The association between retinal measures and MDS-UPDRS III and H-Y staging scores in the total cohort is shown in Table 2. Linear regression analysis revealed a negative correlation between average, temporal quadrant pRNFL thickness and MDS-UPDRS III scores (*p* < 0.05). And the MRT, mRNFL, GCL, IPL, INL, ONL thickness, and TMV were inversely correlated with MDS-UPDRS III scores (all *p* < 0.05). In addition, there was a significant negative correlation between the average and temporal quadrant pRNFL thickness, mRNFL, GCL, IPL, INL thickness, MRT, TMV, and H-Y staging scores. And there was a significant positive correlation between the OPL thickness and H-Y staging scores. However, no correlation was found between other OCT parameters and MDS-UPDRS III, H-Y staging scores (Table 2).

**Table 2:** Relationships between retinal measures and MDS-UPDRS III, H-Y staging, non-motor symptoms in the PD patients.

OCT measures	MDS-UPDRS III		H-Y staging		MMSE		RBDQ-HK		ESS		HRS		HAMD	
	*S*_*β*_	*p*	*S*_*β*_	*p*	*S*_*β*_	*p*	*S*_*β*_	*p*	*S*_*β*_	*p*	*S*_*β*_	*p*	*S*_*β*_	*p*
pRNFL, μm														
Average	**−0.176**	**<** **0.001**	**−0.316**	**<** **0.001**	**0.106**	**0.035**	**−0.130**	**0.010**	−0.006	0.913	0.087	0.085	−0.025	0.625
Superior	−0.033	0.515	−0.084	0.094	**0.136**	**0.007**	0.008	0.867	< 0.001	0.999	−0.004	0.938	−0.010	0.843
Inferior	−0.061	0.229	−0.089	0.075	−0.010	0.841	−0.063	0.214	0.040	0.435	0.054	0.288	0.033	0.522
Nasal	−0.042	0.401	−0.010	0.842	0.005	0.917	−0.025	0.628	0.078	0.125	−0.008	0.867	0.083	0.101
Temporal	**−0.173**	**0.001**	**−0.265**	**<** **0.001**	0.078	0.124	−0.079	0.120	−0.081	0.112	0.034	0.499	−0.090	0.077
macula, μm														
MRT^a^	**−0.330**	**<** **0.001**	**−0.378**	**<** **0.001**	0.074	0.143	**−0.250**	**<** **0.001**	**−0.131**	**0.009**	0.080	0.116	**−0.173**	**0.001**
mRNFL^b^	**−0.322**	**<** **0.001**	**−0.319**	**<** **0.001**	**0.105**	**0.045**	**−0.138**	**0.008**	**−0.123**	**0.019**	0.078	0.138	−0.082	0.116
GCL^b^	**−0.243**	**<** **0.001**	**−0.202**	**<** **0.001**	**0.117**	**0.025**	**−0.170**	**0.001**	**−0.121**	**0.020**	**0.186**	**<** **0.001**	**−0.178**	**0.001**
IPL^b^	**−0.239**	**<** **0.001**	**−0.250**	**<** **0.001**	0.065	0.218	**−0.185**	**<** **0.001**	**−0.169**	**0.001**	**0.103**	**0.049**	−0.077	0.143
INL^b^	**−0.271**	**<** **0.001**	**−0.226**	**<** **0.001**	0.044	0.396	**−0.158**	**0.002**	−0.053	0.311	0.059	0.259	**−0.122**	**0.020**
OPL^b^	−0.009	0.858	**0.235**	**<** **0.001**	−0.021	0.693	0.051	0.326	−0.001	0.980	−0.056	0.288	−0.001	0.978
ONL^b^	**−0.123**	**0.018**	−0.071	0.175	0.012	0.823	−0.100	0.057	0.023	0.657	0.044	0.405	−0.069	0.185
IS/OS^b^	−0.024	0.644	−0.038	0.472	0.011	0.828	0.071	0.178	0.029	0.581	−0.068	0.195	0.023	0.661
outer segment^b^	−0.051	0.334	0.012	0.813	0.007	0.898	−0.027	0.605	0.045	0.388	−0.090	0.085	−0.002	0.971
OPR^b^	−0.076	0.148	−0.059	0.259	**0.114**	**0.029**	−0.080	0.125	0.017	0.751	**0.133**	**0.011**	−0.041	0.431
RPE^b^	−0.028	0.598	−0.059	0.262	0.047	0.374	0.017	0.745	−0.060	0.254	0.049	0.352	0.001	0.983
TMV^b^	**−0.310**	**<** **0.001**	**−0.314**	**<** **0.001**	0.029	0.573	**−0.199**	**<** **0.001**	**−0.143**	**0.005**	**0.154**	**0.002**	**−0.174**	**0.001**

Significant results in bold. A linear regression analysis was used to assess the relationship between OCT measures and MDS-UPDRS III, H-Y staging, MMSE, RBD, ESS, HRS, and HAMD scores, which was adjusted for confounders of age, sex.

*PD* Parkinson’s Disease, *S*_*β*_ Standardized regression coefficient, *OCT* optical coherence tomography, *MDS-UPDRS III* Movement Disorder Society-Unified Parkinson’s Disease Rating Scale III, *H-Y* Hoehn-Yahr, *MMSE* Mini-Mental State Examination, *RBDQ-HK* Rapid Eye Movement sleep behavior disorder (RBD) questionnaire-Hong Kong, *ESS* Epworth sleepiness scale, *HRS* Hyposmia Rating Scale, *HAMD* Hamilton rating scale for depression, *pRNFL* peripapillary retinal nerve fiber layer, *MRT* macular retina thickness, *mRNFL* macular retinal nerve fiber layer, *GCL* ganglion cell layer, *IPL* inner plexiform layer, *INL* inner nuclear layer, *OPL* outer plexiform layer, *ONL* outer nuclear layer, *IS/OS* inner segment/outer segment, *OPR* photoreceptor outer segment/retinal pigment epithelium, *RPE* retinal pigment epithelium, *TMV* total macular volume.

^a^MRT: the full thickness of retina from internal limiting membrane (ILM) to retinal pigment epithelium (RPE).

^b^High-definition optical coherence tomography (HD-OCT) measures (mm) are the average of nine subfields of the Early Treatment Diabetic Retinopathy Study grid centered at the fovea.

### Association between optical coherence tomography parameters and non-motor symptoms

Of all 397 patients with PD, 170 complicated with Rapid Eye Movement sleep behavior disorder (RBD) [RBD questionnaire-Hong Kong (RBDQ-HK) ≥ 18, 42.82%), 121 with excessive daytime sleepiness (EDS) [Epworth sleepiness scale (ESS) ≥ 10, 30.48%], 193 with hyposmia [Hyposmia Rating Scale (HRS) ≤ 22, 48.61%], and 115 with depression [Hamilton rating scale for depression (HAMD) ≥ 8, 28.97%] (Fig. 1).

The linear regression analysis results demonstrated that the average and superior quadrant pRNFL, mRNFL, GCL, and outer segment photoreceptor/retinal pigment epithelium complex (OPR) thickness were positively associated with Mini-Mental State Examination (MMSE) scores (*p* < 0.05). There was an inverse correlation between the average pRNFL, mRNFL, GCL, IPL, INL thickness, MRT, TMV, and RBDQ-HK scores. The mRNFL, GCL, IPL thickness, and MRT and TMV were inversely correlated with ESS scores. In addition, there was a positive association between GCL, IPL, OPR thickness, TMV, and HRS scores. The GCL and INL thickness, and MRT and TMV were negatively correlated with HAMD scores (Table 2).

### The diagnostic performance of retina parameters

**Fig. 4:**
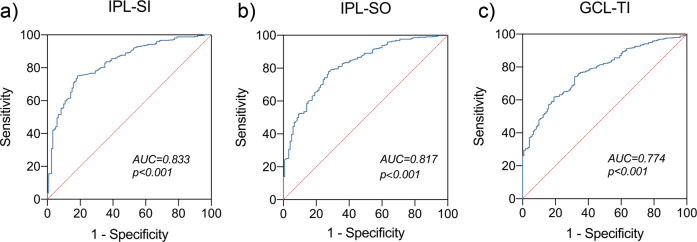
Receiver operating characteristic (ROC) curve for the retinal parameters. Results showed that IPL thickness in the SI subfield (IPL-SI) showed the best diagnostic performance, followed by IPL thickness in the SO subfield (IPL-SO) and GCL thickness in the TI subfield (GCL-TI). IPL inner plexiform layer, GCL ganglion cell layer, SI superior inner, SO superior outer, TI temporal inner.

In the analysis above, we found the single macular intraretinal layer (GCL, IPL, and ONL) were significantly thinner early-on in patients with PD with H-Y I, and further decreased as the H-Y staging score increased. In addition, we observed that the GCL and IPL thicknesses were correlated with both MDS-UPDRS III scores and non-motor symptoms. Based on these results, we carried out further analyses to evaluate the diagnostic performance of the GCL and IPL layers in nine grids of Early Treatment Diabetic Retinopathy Study (ETDRS) by plotting receiver operator characteristic (ROC) curves and calculating the area under the curve (AUC). We found that IPL thickness in the superior inner (SI) subfield (IPL-SI) showed the best diagnostic performance in PD with H-Y I stage *vs* HCs, with a sensitivity and specificity of 75.06% and 81.67%, respectively, and an AUC of 0.833 [95% confidence interval (CI) 0.794 – 0.873]. This was followed by IPL thickness in the superior outer (SO) subfield and GCL thickness in the temporal inner (TI) subfield, with sensitivities and specificities of 77.91% and 72.50%; 61.76% and 80.83%, respectively. The AUCs were 0.817 (95% CI 0.776 – 0.859) and 0.774 (95% CI 0.731 – 0.817), respectively (Fig. 4).

## Discussion

This study evaluated the intraretinal layer thickness changes in all nine ETDRS subfields in patients with PD grouped by the H-Y stage, by utilizing OCT combined with an advanced automated 3D intraretinal layer segmentation software (Iowa Reference Algorithms version 3.8.0). We also correlated OCT measurements with MDS-UPDRS III and non-motor symptoms in the study. We found that IPL-SI thickness can be used as an indicator for the early diagnosis of PD.

The decreasing pRNFL thickness in patients with PD was first reported in 2004^15^, but that study’s sample size was small. Subsequently, many studies have reported pRNFL thinning in patients with PD^10,11,13,16^. Visser et al.^16^ found patients with PD had significantly thinner pRNFL in the temporal retinal quadrant. Ucak et al.^10^ found the mean, superior, and inferior quadrant pRNFL in patients with PD were significantly thinner than those in the control group. Jiménez et al.^11^ found a decrease in the average pRNFL thickness in all four quadrants. However, other studies have reported that the thickness of the pRNFL does not change^9,17,18^. These contradictory results may be due to the limited sample sizes, different OCT devices, and inclusion criteria used in these studies. In the present large-scale study, with rigorous control of patient selection by establishing well-defined inclusion and exclusion criteria, we found the average and temporal quadrant pRNFL thickness were significantly reduced. The comparison of pRNFL between the PD subgroups and HCs group showed that pRNFL in all quadrants in patients with PD with H-Y I did not change significantly, and the temporal quadrant pRNFL was the first to become thinner in patients with PD with H-Y II, followed by a significant decrease in the superior and inferior quadrant pRNFL in patients with PD with H-Y III. We hypothesize that retinal degeneration follows a sequential pattern in PD. In addition, we believe that the pRNFL is not sufficiently sensitive for the early diagnosis of PD. The pRNFL constitutes the axons of the retinal ganglion cell, and, as such, pRNFL thinning in PD is considered to be the degeneration of dopaminergic cells in the GCL resulting from axonal loss^19^. In the present study, temporal quadrant pRNFL thickness was more susceptible to reduction, which was consistent with the results of previous studies^15,16^. Like the axonal loss pattern that occurs in Leber’s hereditary optic neuropathy and dominant optic atrophy, the preferential loss of the temporal quadrant of the pRNFL in PD may be due to mitochondrial dysfunction^20,21^, which was described as an underlying pathophysiological mechanism in PD^22^. Previous studies have shown that the temporal quadrant pRNFL contains the papillomacular bundle, which is especially sensitive to mitochondrial dysfunction^21^.

Our findings were consistent with those of previous studies, showing a significant reduction in TMV and MRT^10,20,23–26^. To explore the specific diagnostic retinal markers in patients with PD, we applied an automated 3D intraretinal layer segmentation software to segment the macula into ten layers and calculated the single-layer thickness. We found a significant thinning in mRNFL, GCL, IPL, INL, and ONL thickness in patients with PD compared to HCs and a significant increase in the OPL thickness. Furthermore, the changes in patients with PD were more pronounced, with higher H-Y staging scores; this suggests that macular thinning correlates with disease progression. Moreover, our results showed that GCL, IPL, and ONL were significantly thinner early in PD with the H-Y I stage. These changes occur in the early stages of PD. Based on these results, macular parameters seemed to show better diagnostic ability than pRNFL. Moreover, in our analysis, we found no thinning in MRT including the full retinal thickness in patients with PD with the H-Y I stage. Thus, we confirmed that total macular thickness was not a reliable marker for PD diagnosis. Dopamine, a key neurotransmitter in the retina, can modulate neural activity and visual function. Previous studies have confirmed that α-synuclein (the main components of Lewy bodies) deposits and dopaminergic cells are limited to the macular inner retina layer (RNFL, GCL, IPL, and INL)^4,27,28^. The loss of dopaminergic amacrine cells and its intraocular injection may reduce macular thickness and TMV^4^. Furthermore, dopaminergic deficiency may contribute to the over-production of glutamate, neuronal death, and cellular dysregulation^29^. Therefore, we can infer that OCT measures may reflect the number of retinal dopaminergic cells; this also provided evidence to support the pathological association between the retina and brain in PD. The most interesting finding in our study was the thicker OPL in patients with PD compared to HCs. The exact cause is not yet fully understood; this can be explained by the hypothesis that the neurodegenerative process of the inner retinal layers triggers compensatory outer retinal thickening^30^. However, our findings require cautious interpretation and replication in other patient cohorts.

Our results are consistent with previous studies investigating the correlation between OCT measurements and MDS-UPDRS in patients with PD. Altintas et al.^26^ found an inverse correlation between foveal retinal thickness and UPDRS scores. Jimenez et al.^11^ reported that there was an inverse correlation between mean pRNFL thickness and UPDRS scores. The negative correlation between retinal thinning and MDS-UPDRS III scores in PD demonstrated in our study may suggest the possibility of dopaminergic depletion in the retina concurrent with that of the basal ganglia pathophysiological process of PD^9^; this presents further evidence for the hypothesis that the brain and retina share a common pathophysiology.

Non-motor symptoms including cognitive function impairment, RBD, EDS, hyposmia, and depression have been considered promising prodromal markers of PD^31^. To our knowledge, no research has attempted to correlate retinal alternations with non-motor symptoms in PD. Our data showed an association between macular inner retinal layer alterations and non-motor symptoms in patients with PD. These findings suggest that macular inner retinal layer thickness is much thinner in patients with PD with non-motor symptoms. The onset of non-motor symptoms in PD is related to aberrant α-synuclein activity in the brain. Previous studies revealed that α-synuclein also aggregates in the inner retinal layer^28^. Pathological α-synuclein may cause damage to synapses and interrupt signal transmission, and ultimately lead to brain neuron death^32^. With PD progression, the macular inner retinal layer thickness is thinner, as confirmed in our findings above; astrocyte endocytosis is impaired; α-synuclein levels increase^33^; and non-motor symptoms become more severe.

In summary, this large-scale study confirmed that the retinal structure was significantly altered in patients with PD in different clinical stages, and that macular inner retinal layer changes occurred during early PD disease. Moreover, we verified that macular IPL-SI thickness may be used as an early diagnosis and disease progression monitoring indicator in patients with PD.

## Methods

### Study population

We selected patients with PD from outpatients and inpatients in the Department of Neurology of the Xiangya Hospital from December 2017 to November 2019. HCs with matching age and sex were recruited from the Health Management Center of the Third Xiangya Hospital. HCs had no history of any eye, nervous, or systemic diseases. A detailed questionnaire-based interview was administered by well-trained researchers in the field of neurology. The following demographic and clinical information were collected: age, sex, age at onset, and diagnosis and duration of disease. The inclusion criteria of the study were: age 50–80 years, BCVA ≥ 0.5, refractive spherical equivalent ≤6.00 D and/or with astigmatism ≤3.00 D, and IOP less than 21 mmHg. The exclusion criteria were: systemic diseases that may affect vision, such as diabetes, uncontrolled hypertension, and ischemic heart disease; eye diseases, history of eye trauma, and any eye surgery; other neurological or mental diseases; and poor HD-OCT image quality (signal strengths <7) and inability to follow OCT procedures due to severe Parkinson’s movement disorder. The study followed the Declaration of Helsinki and was approved by the Clinical Research Ethics Committee of Xiangya Hospital of Central South University. All participants provided written informed consent before being included in our study.

### Assessment of motor symptoms

A total of 397 PD and 427 age- and sex-matched HC participants were enrolled in the current study. The diagnosis of PD was based on the Movement Disorder Society clinical diagnosis criteria^34^, and all patients were clinically stable under current drug treatments. The motor symptoms of patients with PD were assessed using the MDS-UPDRS III^35^ and the H-Y staging scale^36^. A higher score indicates a more severe disease and disability. In the late stages of the disease, especially in patients with pronounced tremors, high-quality OCT scans cannot be obtained. These patients must be excluded and were therefore not included in the study; thus, the H-Y staging of PD in our study ranged from I to III.

### Assessment of non-motor symptoms

The non-motor symptoms of PD include cognitive function decline, RBD, EDS, hyposmia, and depression. Cognitive function was assessed using the Chinese version of MMSE^37^. RBD and EDS were evaluated using the RBDQHK^38^ and ESS^39^, respectively. Olfactory function and depression were assessed by HRS^40^ and HAMD^41^, respectively.

### Assessment of eyes

All participants underwent a comprehensive eye examination, including BCVA evaluation using a Snellen chart, measurement of Goldmann applanation IOP, fissure lamp biomicroscopy, fundus photography, and HD-OCT examination. These examinations were performed by an ophthalmologist (XB) in a blinded manner.

### High-definition optical coherence tomography examination

Following a detailed eye examination, a random eye was scanned to obtain the pRNFL and macular parameters. Briefly, two protocols for random eyes were performed on each participant using Cirrus HD-OCT 5000 (Carl Zeiss Meditec, Inc., Dublin, CA, USA). The “Optic Disc Cube 200 × 200” scan protocol included a 3.4 mm diameter circular scan consisting of 256 A-scans around the optic nerve head. The average pRNFL thickness and pRNFL thickness in the four quadrants (superior, inferior, nasal, and temporal) were measured according to the “Optic Disc Cube 200 × 200” protocol. The “Macular Cube 512 × 128” protocol incorporated six consecutive 6 mm line scans centered on the fovea, each containing 128 equally spaced transverse axial scans taken in a single session of 1.92 s. The automatic analysis algorithm displayed the TMV and MRT of the nine regions corresponding to the ETDRS. The ETDRS area includes a center circle of 1 mm (the fovea), an inner circle, and an outer circle, with diameters of 3 mm and 6 mm respectively. The inner circle and the outer circle were divided into four quadrants: superior, nasal, inferior, and temporal. No manual correction was performed on the OCT images in the present study, but the correct segmentation and image quality of all scans were assessed. Only images with a signal strength ≥ 7 were analyzed, and images with poor quality before data analysis were rejected. All scans were performed by experienced operators who were blinded to the diagnosis of PD.

### Intraretinal layer segmentation

Macular OCT images were imported into the Iowa Reference Algorithms version 3.8.0 software (Retinal Image Analysis Lab, Iowa Institute for Biomedical Imaging, Iowa City, IA, USA) for automated intraretinal layer segmentation^42^. Previous studies have reported that the algorithms demonstrated higher reliability and reproducibility^14,43^. In brief, this algorithm delineated 11 optical surfaces from the internal limiting membrane to the retinal pigment epithelium and automatically delimited the following ten retinal layers: mRNFL, GCL, IPL, INL, OPL, ONL, IS/OS (inner segment/outer segment) layer, outer segment, OPR, and RPE. The thickness of the ten retinal layers was calculated for each of the nine ETDRS subfields. We analyzed the mean thickness of the ten retinal layers in all nine ETDRS grids on all scans. Proper segmentation of all images was performed by an image analyst masked to the clinical diagnosis.

### Statistical analysis

Statistical analysis was performed using SPSS version 25.0 (SPSS Inc., Chicago, IL). The Kolmogorov-Smirnov test was used to determine the normality of the data. The characteristics of the participants are summarized as the mean ± standard deviation or the median (25% percentile, 75% percentile). We used the Pearson’s *χ*2 test and Student’s t-test to evaluate the differences in demographic profiles between patients with PD and the HCs groups. The general linear model was used to compare OCT measurements between the PD and HCs groups with adjustment for confounders (age, sex, IOP, BCVA). Similar analyses were performed for comparisons between each PD subgroup versus HCs. Linear regression analysis was performed to assess whether an association existed between retinal parameters, and motor and non-motor symptoms. The non-parametric test was used when data were not normally distributed. The AUC was calculated to evaluate the diagnostic performance of the OCT parameters. *p* < 0.05 was considered statistically significant.

## Data Availability

All data that support the findings of the current study are available from the corresponding authors upon reasonable request.
